# Pathophysiology of Vascular Stenosis and Remodeling in Moyamoya Disease

**DOI:** 10.3389/fneur.2021.661578

**Published:** 2021-09-03

**Authors:** Brandon M. Fox, Kirsten B. Dorschel, Michael T. Lawton, John E. Wanebo

**Affiliations:** ^1^Department of Neurosurgery, St. Joseph's Hospital and Medical Center, Barrow Neurological Institute, Phoenix, AZ, United States; ^2^Medical Faculty, Heidelberg University Medical School, Ruprecht-Karls-Universität Heidelberg, Heidelberg, Germany

**Keywords:** angiopathy, cerebrovascular, moyamoya, stenosis, stroke

## Abstract

Moyamoya disease (MMD) and moyamoya syndrome (MMS) are progressive vascular pathologies unique to the cerebrovasculature that are important causes of stroke in both children and adults. The natural history of MMD is characterized by primary progressive stenosis of the supraclinoid internal carotid artery, followed by the formation of fragile collateral vascular networks. In MMS, stenosis and collateralization occur in patients with an associated disease or condition. The pathological features of the stenosis associated with MMD include neointimal hyperplasia, disruption of the internal elastic lamina, and medial attenuation, which ultimately lead to progressive decreases in both luminal and external arterial diameter. Several molecular pathways have been implicated in the pathophysiology of stenosis in MMD with functions in cellular proliferation and migration, extracellular matrix remodeling, apoptosis, and vascular inflammation. Importantly, several of these molecular pathways overlap with those known to contribute to diseases of systemic arterial stenosis, such as atherosclerosis and fibromuscular dysplasia (FMD). Despite these possible shared mechanisms of stenosis, the contrast of MMD with other stenotic pathologies highlights the central questions underlying its pathogenesis. These questions include why the stenosis that is associated with MMD occurs in such a specific and limited anatomic location and what process initiates this stenosis. Further investigation of these questions is critical to developing an understanding of MMD that may lead to disease-modifying medical therapies. This review may be of interest to scientists, neurosurgeons, and neurologists involved in both moyamoya research and treatment and provides a review of pathophysiologic processes relevant to diseases of arterial stenosis on a broader scale.

## Introduction

Moyamoya disease (MMD) and moyamoya syndrome (MMS) are cerebrovascular pathologies that are characterized by progressive stenosis and eventual occlusion of the intracranial internal carotid artery (ICA) and its proximal middle cerebral artery (MCA) and anterior cerebral artery (ACA) branches. This stenosis and occlusion cause ischemia and subsequent small vessel collateralization of the brain parenchyma that is supplied by the affected arterial segments. On cerebral angiography, these collateral vascular networks are visible as regions of contrast blush in the early arterial phase, and this distinctive appearance led to the name “moyamoya,” which is Japanese for a puff of smoke (1).

When the term moyamoya is used alone, it refers to the characteristic pathologic findings of stenosis and collateralization. Moyamoya disease refers to primary moyamoya and includes this presentation in individuals who harbor the genetic risk alleles. Moyamoya syndrome refers to moyamoya that occurs in the setting of a known associated disease, condition, or exposure (2). Previously, unilateral moyamoya was classified as MMS, but this categorization has fallen out of favor due to mounting evidence that unilateral pathology frequently progresses to bilateral disease (3–8).

The most common presenting symptom of MMD and MMS is ischemic stroke, which occurs secondary to decreased perfusion downstream from moyamoya stenosis. Additionally, moyamoya frequently presents with transient ischemic attacks (TIAs), which are often recurrent, and less commonly presents with seizures or headaches (9). Intracranial hemorrhage also occurs in MMD and MMS due to the rupture of vessels in fragile collateral networks or associated aneurysms (10), and importantly, hemorrhage occurs as the presenting symptom more frequently as patient age increases (11). In 2018, Funaki et al. demonstrated that choroidal collaterals may be a bleeding source with a high risk for hemorrhagic recurrence and a predictor of recurrence in hemorrhagic MMD (12). Preliminary results obtained in 2019 by Funaki et al. (13) suggest that the presence of choroidal collaterals may affect the risk of *de novo* hemorrhage in nonhemorrhagic hemispheres; however, these results remain subject to verification in larger studies.

Moyamoya disease and MMS are relatively rare and occur with varying incidence in populations throughout the world. Incidence rates are highest in eastern Asia, and specifically Japan, where its incidence is reported at 0.35 per 100,000 individuals (14). By contrast, the overall incidence reported from the United States is 0.09 per 10,000 individuals, with the highest incidence occurring in Asian Americans (0.28/100,000), followed by African Americans (0.13/100,000), Caucasian Americans (0.06/100,000), and Hispanic Americans (0.03/100,000) (15). The age of onset occurs with a bimodal distribution, with an initial peak in the first decade of life and a second peak in the fifth decade, and studies suggest that the incidence in adults may be increasing (14, 16, 17).

Genetic risk factors may be involved in the development of MMD, and this idea is supported by differences in the incidence of MMD in different populations. Further support for inherited risk was provided by familial cases and a high concordance between monozygotic twins (18). However, incomplete penetrance and discordance have led some to suggest the necessity of an environmental “second hit” or possible epigenetic contribution (19, 20). Genetic linkage analysis from sequencing data identified MMD risk alleles and ultimately led to the identification of *RNF213* as the first known susceptibility gene for MMD (21, 22). Despite this progress, the definitive mechanisms through which mutant *RNF213* contributes to the initiation or progression of moyamoya pathology remain elusive. In 2012, Miyawaki et al. (23) performed a case-control study at a single hospital analyzing the *RNF213* variant c.14576G>A in patients with non-MMD intracranial major artery stenosis/occlusion (ICASO). The authors found an association between c.14576G>A and non-MMD ICASO. They therefore advocate screening for the c.14576G>A variant in ICASO patients. In 2013, this group performed a case–control study, in which they investigated the occurrence rate of the c.14576G>A variant in 323 ICASO patients (24). They hypothesized that a subset of patients with distinct ICASO phenotypes may have a common genetic variant, *RNF213* c.14576G>A, suggesting that the *RNF213* c.14576G>A variant may be a high-risk allele for ICASO. In 2015, this group performed a study in 78 MMD patients and nine non-atherosclerotic MMS patients demonstrating the absence of the *RNF213* c.14576G.A genetic variant in nonatherosclerotic MMS patients (25). The research group hypothesized that identification of this genetic variant could be used to help clarify the pathophysiology of vascular stenosis in the terminal ICA and associated moyamoya vessels.

Since MMD was first described in 1957, research efforts have significantly increased our understanding of its epidemiology and pathobiology. Despite this increased knowledge, medical treatments do not reverse or prevent progression of the primary disease process. In addition, definitive therapy with revascularization, although effective in reducing stroke risk and symptoms, has no effect on the progression of arterial stenosis (26, 27). Current moyamoya treatment is hindered by the lack of understanding of both the pathology process that initiates stenosis and the key signaling pathways that drive progression. In this review, we examine the unique arterial stenosis that occurs in moyamoya and explore the current state of knowledge related to its pathological characteristics, its molecular mechanisms, and the imaging methods utilized for its detection.

## Methods

References were identified by use of a comprehensive, systematic computerized literature search from January 1, 1957, through January 20, 2021, performed by the authors, using the PubMed, EMBASE, BIOSIS Previews, and Medline databases and various combinations of the key words “angiopathy,” “cerebrovascular,” “moyamoya,” “stenosis,” and “stroke.” Relevant articles on MMD and supplemental basic science articles almost exclusively published in English were included. Reference lists of relevant articles were reviewed for additional references, and 165 articles were included in the final manuscript. As some aspects of MMD and MMS have been studied in greater detail compared to others, several topics receive additional attention. In spite of major progress in research in the field of MMD in recent years, the literature in great part remains descriptive. Continued collaborative clinical and basic research is fundamental to further elucidate the pathophysiology of MMD, which may lead to an increasingly differentiated diagnosis and disease-modifying treatment strategies.

## Pathologic Characteristics of Arterial Stenosis in Moyamoya

The arterial stenosis of moyamoya is the primary process that sets in motion the cascade of pathological adaptations and complications that encompass MMD and MMS. Isolated stenosis without evidence of collateral vessel formation occurs first and is defined as the first of six stages in the progression of the disease (1). In most moyamoya patients, stenosis occurs in the bilateral proximal portions of the anterior cerebral circulation and involves the terminal supraclinoid ICA and the proximal MCA and ACA (1, 28). The posterior circulation is spared in most moyamoya patients, despite possessing intracranial arteries with similar caliber and proximity to affected segments of the anterior circulation. Nevertheless, involvement of the posterior circulation can occur in moyamoya (29), and studies have demonstrated posterior circulation disease in approximately one-third of pediatric and adult patients (30–33). When present, stenosis of the posterior circulation typically affects the posterior communicating artery and the P2 segment of the posterior cerebral artery (PCA), while sparing the basilar artery and P1 segment of the PCA (34). Thus, posterior involvement is likely an extension of the stenotic process from the anterior circulation and only rarely arises independently from the proximal posterior circulation, as characterized by basilar artery and P1 segment involvement. The unique properties of posterior circulation moyamoya have led investigators to suggest an embryologic basis for susceptibility of the arterial segments to develop moyamoya (34, 35). Importantly, several lines of evidence suggest that the involvement of the posterior circulation in MMD is associated with a poor prognosis (36–38). Stenosis of extracranial vessels in association with intracranial moyamoya is uncommon, but several studies have demonstrated associated stenosis of the renal artery in a minority of patients (39, 40). In addition, a few studies have demonstrated associated stenosis of external carotid artery branches, coronary arteries, and abdominal arteries (41, 42). However, other studies have specifically looked for involvement of branches of the external carotid artery or intraabdominal arteries and have failed to demonstrate stenosis in these segments (40, 43). Despite these exceptions, the restriction of moyamoya stenosis to such a limited and specific anatomic region remains one of the most fundamental questions surrounding its pathology, as other forms of medium to large artery stenosis, such as atherosclerosis and fibromuscular dysplasia (FMD), are typically far more widespread throughout the arterial tree.

**Figure 1:**
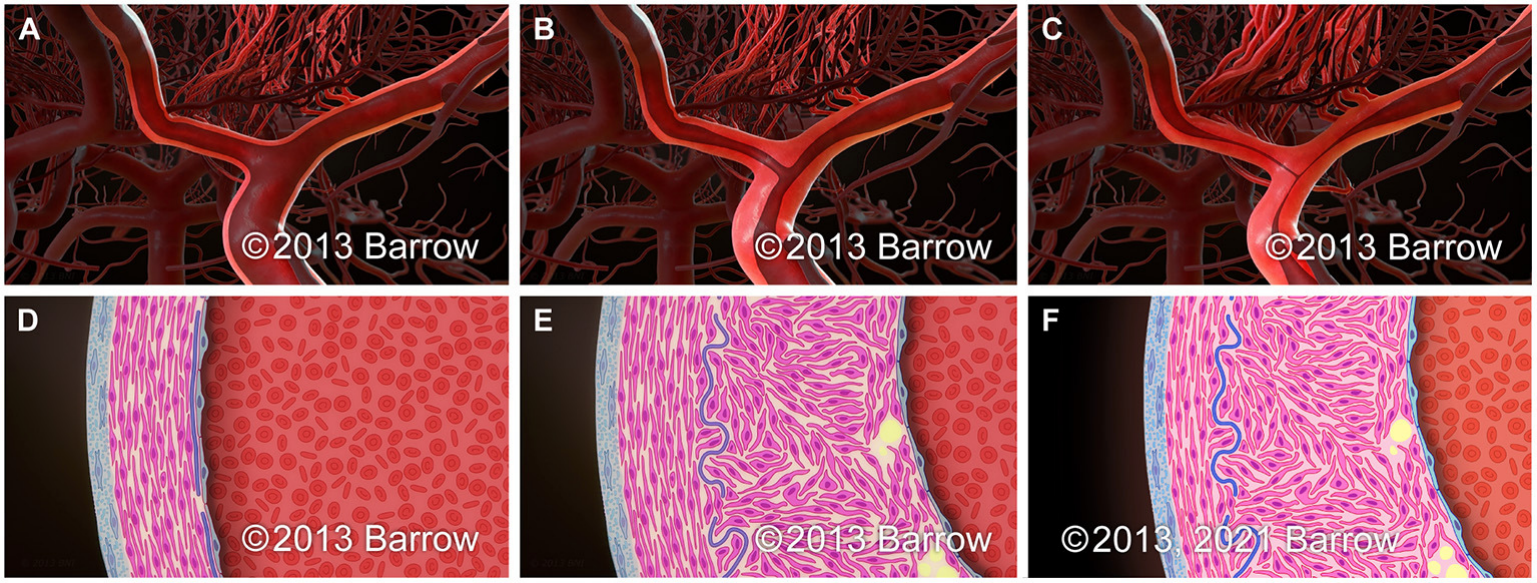
Schematic representation of the paradigmatic progression pattern of arterial stenosis in moyamoya on the macroscopic **(A–C)** and microscopic **(D–F)** scale. **(A,D)** Normal terminal ICA. **(B,E)** Early in the progression of moyamoya, intimal hyperplasia leads to the development of luminal stenosis without a significant change in the outer diameter of the arterial segment. **(C,F)** As luminal stenosis continues to progress in moyamoya, attenuation of the medial layer of the artery leads to a reduction in the outer arterial diameter. *Used with permission from Barrow Neurological Institute, Phoenix, Arizona*.

The histopathologic characteristics of stenosis in moyamoya represent a collection of pathologic processes that, in addition to the gross pathologic features described above, define moyamoya as a unique form of arterial stenosis. The primary cause of moyamoya stenosis is a concentric fibrocellular hyperplasia of the intima (44, 45). This hyperplasia is characterized by a proliferation of smooth muscle cells and extracellular matrix within the intima that leads to progressive intimal thickening (Figure 1) (46–49). In addition, the internal elastic lamina is altered such that it is frequently wavy, duplicated, and sometimes disrupted (45, 50–52). In contrast to the significant intimal thickening that occurs in moyamoya, the tunica media becomes progressively thinner as moyamoya progresses (46). This thinning occurs to the degree that, despite intimal expansion, the outer vessel diameter decreases in association with luminal stenosis (53, 54), and as moyamoya advances over time, the outer diameter progressively decreases (Figure 1) (55). In 2016, Yamamoto et al. suggested that MMS may comprise two distinct pathological subgroups, a constrictive subgroup and a nonconstrictive subgroup. Patients in the constrictive subgroup of MMS may show marked constrictive vascular changes and limited intimal hyperplasia. Patients in the non-constrictive subgroup of MMS may show profuse fibrosis (56). Luminal thrombi and microthrombi are frequently observed in autopsy specimens from moyamoya patients and are frequently associated with regions of intimal hyperplasia and stenosis (57). These thrombi contribute to arterial occlusion in moyamoya and are thought to be related to intimal pathology (2, 57).

Like moyamoya, atherosclerosis causes stenosis with intimal expansion and intimal smooth muscle cell hyperplasia. However, atherosclerosis is marked by characteristic inflammatory infiltration and lipid accumulation that do not typically occur in moyamoya (48). Furthermore, as luminal stenosis progresses in atherosclerosis, outward vascular remodeling occurs, with increased outer vessel diameter (58, 59), in contrast to the decreased outer diameter that occurs in moyamoya. Another form of arteriopathy, the intimal fibroplasia subtype of FMD, is characterized by intimal expansion in the absence of inflammatory cell infiltration or lipid accumulation (60, 61). Like moyamoya, it causes intimal extracellular matrix expansion and abnormalities of the internal elastic lamina, but the intimal fibroplasia subtype of FMD is not characterized by hyperplasia of the intima as is seen in moyamoya (62). However, pediatric patients with FMD can also present with stroke and renal artery stenosis (62, 63). These shared pathologic features highlight the need for future research to better understand the complex relationship between these two entities.

## Vascular Remodeling in Moyamoya Disease

The vascular wall pathology in MMD involves thickening of the intima without infiltration of inflammatory cells or considerable disruption of the internal elastic lamina (51, 64). Compared to typical findings in atherosclerosis, macrophage infiltration into the subintimal layer, and lipid pool accumulation are not characteristic features of moyamoya (64, 65). In contrast, a study by Masuda et al. (66) of autopsy specimens obtained from MMD patients showed proliferation of smooth muscle cells, the presence of T cells, and infiltration of macrophages into the vascular wall. In MMS associated with neurofibromatosis type I (NF-1), inflammatory cells may infiltrate the area surrounding the lesion of the arteriopathy, indicating that some types of MMS may be related to inflammation (64, 66). Kaku et al. (54) showed a reduction in both the inner and outer diameter of the ICA in the course of the moyamoya remodeling process, in contrast to the pathogenesis of atherosclerosis, which shows an outward remodeling pattern. These authors also found no reduction of the outer diameter of the circle of Willis in an MMS subgroup, and arterial shrinkage was insignificant (54, 64). Moyamoya vascular shrinkage may be detected specifically in the horizontal MCA (M1) section (64, 67). According to Yamamoto et al. (56), MMS pathology may include two distinct subgroups: a nonconstrictive subgroup as well as a constrictive subgroup. A portion of one subgroup of MMS may show excessive fibrosis. Another subgroup of MMS may show limited intimal hyperplasia and vascular constrictive changes (64). In 2015, Mikami et al. (67) showed an overall reduction in the size of the outer diameter of all vessels around C1 in MMD patients. Similar to a negative constrictive remodeling pattern, the MCA diameter was shown to be significantly smaller in patients with disease progression (67). In 2019, Choi et al. (68) used high-resolution MRI to compare plaque characteristics and the vascular remodeling pattern and hemodynamic changes related to intracranial plaques depending on the presence or absence of the *RNF213* p.Arg4810Lys variant in patients without moyamoya and demonstrated that, in these patients, the *RNF213* variant was associated with smaller outer vessel diameters in the distal ICA, proximal MCA, and basilar artery (68).

## Moyamoya Syndrome, Pediatric Moyamoya Symptomatology, and Associated Molecular Pathways

### Moyamoya Syndrome

Moyamoya disease is idiopathic and occurs as a primary pathologic process. Conversely, MMS occurs in patients with a known associated condition, disease, or exposure that places the patient at significantly higher risk of developing moyamoya. The most frequent moyamoya-associated conditions are sickle cell disease, Down's syndrome, NF-1, and exposure to cranial radiotherapy, which are present in 10–20% of moyamoya cases (69). Less frequently associated conditions include: Alagille syndrome, Costello syndrome, microcephalic osteodysplastic primordial dwarfism type II, Noonan syndrome, Seckel syndrome, hyperthyroidism, and many others (2, 70–78). Importantly, moyamoya-associated conditions have the potential to inform the investigation into the mechanisms that may be important for the development of moyamoya. The genetic basis of many of these associated conditions is well-understood and, thus, provides important hints as to the importance of various signaling pathways in the development of moyamoya.

### Moyamoya Syndrome and Associated Signaling Pathways

By identifying genes involved in MMD pathogenesis and several monogenic MMSs, investigators have associated various molecular pathways with MMD pathophysiology, including the Ras–Raf–mitogen-activated protein kinase (MEK)–extracellular-signal-related kinase (ERK) signaling pathway (NF-1, Noonan syndrome, Costello syndrome), the neurogenic locus notch homolog protein (Notch) signaling pathway (Alagille syndrome), the nitric oxide (NO)-soluble guanylyl cyclase (sGC) signaling pathway [Guanylate cyclase 1 soluble subunit alpha-3 (GUCY1A3)], and pathways involved in genomic stability [Lys-63-specific deubiquitinase BRCC36 [BRCC3], pericentrin [*PCNT*], and other genes involved in dwarfism] (20) (See the Supplementary Table 1 for definitions of gene symbols, proteins, and additional terminology).

The Ras-Raf-MEK-ERK signaling pathway has been shown to be associated with NF-1 (79, 80). Loss-of-function mutations in *NF1* (17q11.2) lead to absent neurofibromin expression, which in turn may activate the Ras-Raf-MEK-ERK signaling pathway, leading to an increased mitogenic signaling (79, 80). In Noonan syndrome, gain-of-function mutations in *PTPN11* (12q24.13), *NRAS* (1p13.2), *SOS1* (2p22.1), *RAF1* (3p25.2), *BRAF* (7q34), *KRAS* (12p12.1), and *MAP2K1* (15q22.31) genes may activate the Ras-Raf-MEK-ERK signaling pathway (20, 81–84).

Costello syndrome is encoded by *HRAS* (11p15.5) and *KRAS* (12p12.1), and gain-of-function mutations influencing p.Gly13 or p.Gly12 may cause germline activation of *HRAS*, and thereby lead to dysregulation of the Ras-Raf-MEK-ERK signaling pathway (20, 85, 86).

Defects in the Notch signaling pathway are associated with vasculopathy. Alagille syndrome is caused by loss-of-function mutations in either *JAG1* (20p12.2) or *NOTCH2* (1p12-p11) (20, 87–96).

Genomic stability signaling pathways are associated with congenital dwarfing syndromes. *PCNT* (21q22.3) loss-of-function mutations are linked to deficient pericentrin cell cycle progression in microcephalic osteodysplastic primordial dwarfism type II. In Seckel syndrome, *CEP63* (3q22.2), *ATR* (3q23), *CENPJ* (13q12.12), *NIN* (14q22.1), *CEP152* (15q21.1), and *RBBP8* (18q11.2) are associated with cycle cell progression, centrosomal function, and DNA repair (20, 71, 97–99).

BRCC3/MTCP1 is associated with MMD pathogenesis. *BRCC3* (Xq28) is potentially involved in DNA repair alteration. Absent BRCC3 expression inhibits angiogenesis of trunk intersegmental vessels in the zebrafish. MTCP1 may enhance phosphorylation and activation of *AKT1* and *AKT2* (20, 100–102).

The nitric oxide-soluble guanylyl cyclase-cyclic guanosine monophosphate (NO-sGC-cGMP) signaling pathway is a key signal transduction pathway associated with controlling vascular smooth-muscle relaxation, vascular tone, and vascular remodeling. It is hypothesized that homozygous loss-of-function mutations in *GUCY1A3* (4q32.1) may be involved in an alteration of the NO pathway in vascular smooth muscle cells (SMCs), potentially leading to aberrant vascular remodeling at bifurcation and curvature regions, such as the ICA bifurcation (20, 103).

### Moyamoya Syndrome and Associated Autoimmune and Inflammatory Diseases

Moyamoya Syndrome in association with inflammation occurs with a frequency of 0.54–1.5% in pediatric MMD patients compared to a frequency of 1.7–4.7% in adult MMD patients (64, 104). In 1983, Suzuki et al. (105) analyzed the incidence of infections in MMD patients. They found that 61.6% of adult MMD patients and 82.6% of pediatric MMD patients suffered from infections of the face and head including both infections of unknown origin and maxillary sinusitis, otitis media, and tonsillitis (64). According to the Japanese national survey by Hayashi and colleagues (106), diseases other than atherosclerosis constitute 17.2% of all inflammatory diseases associated with MMS. For common autoimmune disorders related to MMS, meningitis constitutes 2.2%, hyperthyroidism constitutes 7.5%, and other autoimmune disorders constitute 17.2%. Less common autoimmune disorders associated with MMS may include polyarteritis nodosa, dermatomyositis, Addison's disease, Sjögren's syndrome, Kawasaki's disease, acute limbic encephalitis with anti-LGI1 antibody, antiphospholipid antibody syndrome, granulomatosis with polyangiitis, myasthenia gravis, systemic lupus erythematosus, multiple sclerosis, systemic sclerosis, primary systemic vasculitis, rheumatoid arthritis, polymyositis, and thyroiditis (64, 104, 106, 107). It may be difficult to distinguish the mechanisms associated with a distinct autoimmune disorder and from those associated with MMS because of the complexity of the differentiation between causality and correlation (64). In summary, a correlation between chronic systemic inflammation caused by an autoimmune response and MMS may be hypothesized (64).

Moyamoya Syndrome concomitant with hyperthyroidism is seen predominantly in females and may be associated with ischemia, a comparatively advanced age of disease onset, and faster disease progression compared to MMS (64, 108–111). Ischemia may worsen thyroid functioning. Ischemic symptoms caused by thyrotoxicosis were ameliorated after hyperthyroidism was controlled (64, 109). Increased thyroid hormone may cause MMS progression through an increase in vascular sensitivity to the sympathetic nervous system (64, 112). A T-cell-mediated autoimmune response may also advance MMS pathogenesis. Consequently, immunosuppressive treatment before surgery and/or treatment of a hormonal abnormality may be effective in MMS management related to autoimmune disorders (64, 113, 114). Tendler et al. (115) indicated that immunological stimulation in hyperthyroidism and vascular dysregulation and cellular proliferation in MMS may share a pathomechanism involving T-cell dysregulation. This pathomechanism mentioned above involves both vascular morphological alterations and vascular sensitivity (64, 116). In context with morphological alterations, surgical procedures including surgical revascularization may be utilized (64).

Moyamoya Syndrome caused by meningitis is rare, constituting approximately 2.2% of all disorders classified as MMS (64, 106). The associated infection may be caused by various pathogens, including *Hemophilus influenzae* (117, 118), *Streptococcus pneumoniae* (113, 119, 120), β-hemolytic group A *Streptococcus* (121), *Mycobacterium tuberculosis* (122–125), *Propionibacterium acnes* (126), *Leptospira* (127), *Mycoplasma pneumoniae* (128), *Neisseria meningitidis* with cytomegalovirus (129), neurosyphilis with human immunodeficiency virus (HIV) (130), HIV (131–133), Epstein–Barr virus (64, 129), varicella-zoster virus (134), and measles virus (135). Vascular complications related to meningitis usually become clinically manifest during the 2 weeks after disease onset (64, 136, 137). In MMS associated with meningitis, late-onset morphological alteration of the circle of Willis may be observed (113, 117–119, 123, 125). In addition, an increase in autoimmune antibodies may indicate an autoimmune trigger for the onset of MMS (64, 113). Liu et al. (138) found that the cerebrospinal fluid of MMS patients showed an immune response to leptospirosis and that MMS appears to be related to immune reactive vasculitis (64). In 2018, Takahashi et al. reported a case of MMS in familial MMD 9 years after the patient had nonherpetic acute limbic encephalitis (64, 139). The patient tested positive for anti-LGI1 antibodies, and the authors hypothesized that inflammation caused by an autoimmune disease process may have contributed to MMS progression. Consequently, chronic inflammation caused by an autoimmune disease process may lead to MMS in the course of acute inflammation. In addition, acute systemic inflammation may be related to MMD pathogenesis (64).

### Pediatric Moyamoya Symptomatology and Associated Mechanisms

Pediatric MMD patients presenting with headache or fatigue as the primary symptom may be misdiagnosed as having hypochondriasis or as experiencing a developmental deficit, or they may be simply dismissed as being “lazy.” Lack of appropriate treatment in these patients may lead to severe ischemic stroke. General practitioners and physicians who do not routinely encounter MMD should be encouraged to consider a diagnosis of MMD in patients presenting with minor symptoms such as headache, fatigue, and school refusal.

Symptoms of MMD may be ascribed to changes in blood flow due to ICA stenosis. Two major etiologic categories of symptoms may be distinguished, those involving the consequences of compensatory mechanisms in response to ischemia, such as headache from dilated transdural collateral vessels and hemorrhage from fragile collateral vessels, and those caused by cerebral ischemia such as transient ischemic attacks, stroke, and seizures. Variations in the degree of arterial involvement, ischemic cortex areas, progression of stenosis, and response to a reduction in blood supply may help to clarify the broad range of symptoms in MMD (2). In Asian populations, 68% of children with MMD present with ischemic strokes or TIAs, and 2.8% present with hemorrhage (2, 140). Most children and adults with MMD in the United States present with symptoms of ischemia, with a rate of hemorrhage of 20.0% among adults compared to a rate of 2.8% among children (2, 140, 141). An increased frequency of completed strokes is evident in pediatric MMD. Presumably, children's inability to accurately report their symptoms may hinder a timely diagnosis and lead to an increased probability of completed strokes (2, 142). The symptoms of cerebral ischemia in patients with MMD are related to the areas of the brain whose blood flow is supplied by the ICAs and MCAs, specifically, the temporal, parietal, and frontal lobes. The predominant symptoms are cognitive impairment, aphasia, dysarthria, and hemiparesis (2, 140). Additional symptoms include syncope, visual deficits, seizures, and personality changes, the latter of which may be easily confused with a psychiatric disorder (2, 143). Common childhood actions, including crying with hyperventilation, may precipitate a stroke or TIA. Signs of cerebral ischemia may be temporary or permanent, and may be caused by induction of anesthesia for surgery or by exertion. A hypothesized mechanism precipitating strokes and TIAs in these patients is that the cortical vasculature, which is assumed to be fully dilated under the conditions of chronic ischemia, may constrict in response to a decrease in the partial pressure of carbon dioxide, this restriction may be caused by hyperventilation, eventually resulting in a reduction of cerebral perfusion (2, 144). Ischemic symptoms may also be precipitated by dehydration (2). Intracranial hemorrhage, which is frequently encountered in adult MMD patients, is equally evident in pediatric MMD patients (2, 140, 145). Intracranial hemorrhage occurs in the subarachnoid space, in the ventricles, and in the brain parenchyma, commonly in the basal ganglia area. In the past, intracranial hemorrhage has been ascribed to the rupture of moyamoya-associated fragile collateral vessels in the course of progressive ICA stenosis (2, 146, 147). An additional cause of hemorrhage in these patients may be alternating circulatory patterns at the base of the brain, which have been implicated in the emergence of intracranial aneurysms; these aneurysms are usually located at the apex of the basilar artery or in the PCA, which are common locations of increased shear stress in MMD (2, 148, 149). Headache is a predominant presenting symptom in MMD patients. In their 2005 review article, Seol at al. hypothesized that dilatation of leptomeningeal and meningeal collateral vessels may lead to the stimulation of dural nociceptors (2, 150). Characteristically, headache is refractory to therapy and resembles migraine in quality; despite effective surgical revascularization, headache may persist in as much as 63% of MMD patients (2, 150). Headache symptoms may abate within 1 year after surgery for MMD in some moyamoya patients, suggesting a potential regression of basal collaterals. Dilated moyamoya-related collateral vessels in the basal ganglia may be associated with the emergence of choreiform movements in pediatric MMD patients (2, 140, 151). In a study of pediatric MMD patients presenting with choreiform movements, 8 of 10 pediatric MMD patients experienced resolution of this symptom 1 year after revascularization surgery, suggesting that this surgical procedure appears to be effective in reducing the number of moyamoya-related collateral vessels in the basal ganglia (2). Another sign of MMD is the “morning glory disk,” which is an optic disk enlargement associated with retinovascular aberrations; this sign may be evaluated by vascular imaging (2, 152).

Kawabori et al. (153) studied 29 pediatric MMD patients, hypothesizing that persistent disturbance of cerebral hemodynamics may be associated with severe headache in pediatric MMD. They found that encephalo-duro-myo-arteriopericranial synangiosis and STA-MCA anastomosis may be effective surgical procedures to causally treat headache by supplying collateral blood flow to the operated hemispheres. They advocate close follow-up because of the potential for the development of postoperative headaches in these patients (153). In 2019, Riordan et al. (154) studied 59 pediatric MMD patients who had undergone surgical revascularization by pial synangiosis more than 20 years previously and found that pial synangiosis may confer long-term protection against stroke in pediatric MMD. A history of cranial irradiation was ascertained in one patient with a late stroke and in four out of five deceased patients. The authors conclude that, in the absence of significant preoperative comorbidities and neurological deficits, revascularization surgery appears to be safe and effective (154). In 2019, Kazumata et al. (155) performed a prospective, observational, single-center study in 21 pediatric MMD patients [mean age 10 (3.0) years, range 5–14 years], hypothesizing mild cognitive dysfunction caused by cerebral hypoperfusion through the association of a reduced regional cerebral blood flow (rCBF) in the left dorsolateral prefrontal cortex (DLPFC) and medial frontal cortex with a reduced processing speed index (PSI), perceptual reasoning index (PRI), and full-scale intelligence quotient (FIQ). However, they found that average intellectual ability was not reduced in pediatric MMD patients. They also found that the angiographic severity of the disease, ischemic symptoms, family history, and patient age at disease onset were not associated with inadequate cognitive performance (155). In 2019, Lee et al. (156) studied 131 surgically treated pediatric MMD patients to evaluate the risk factors and prevalence of hypertension. They demonstrated a high prevalence of hypertension in pediatric MMD patients. Consequently, they suggest that sufficient efforts should be made to achieve BP reduction in pediatric MMD patients to prevent subsequent vascular events (156).

## Moyamoya Imaging

From its discovery and definition, moyamoya has been a disease process that is diagnosed and classified based on vascular imaging studies. Recent advances in moyamoya imaging have furthered our understanding of the natural history of arterial stenosis in moyamoya and provide important insights into patient responses to therapeutic interventions.

### Moyamoya Imaging Methods and Clinical Evaluation

In 2013, Kim et al. performed a high-resolution MRI (HR-MRI) study in patients with an MCA occlusion stroke that were angiographically confirmed to be 12 patients with MMD and 20 patients with intracranial atherosclerotic disease (ICAD). Consistent with previous pathological reports, the research group showed that MMD may be associated with smaller, concentric occlusive lesions that are rarely enhanced compared to symptomatic ICAD, suggesting that HR-MRI may assist in the evaluation of pathological changes in the vascular wall and in differentiating ICAD from MMD (157). In 2015, Kim et al. performed a case-control study in MMD patients and normal controls using magnetic resonance angiography (MRA) and computational fluid dynamics. The research group demonstrated significant morphological differences in the intracranial-extradural ICA of MMD patients. These differences may affect the hemodynamics around the ICA bifurcation. Whether these hemodynamic changes are the result or a cause of the ICA bifurcation stenosis remains to be elucidated (158).

Ladner et al. (159) proposed a scoring system for the degree of severity of moyamoya, Prior Infarcts, Reactivity, and Angiography in Moyamoya Disease (PIRAMD), which uses a combination of conventional angiography and non-invasive structural and hemodynamic 3 Tesla MRI parameters. These parameters potentially provide a measurement of the hemodynamic degree of severity of MMD, which has proved to correlate well with symptoms, and which may assist in evaluating intervention response and guiding management decisions. Limitations of this study, which was performed in 11 healthy control volunteers and 25 MMD patients with angiographically confirmed moyamoya, include its retrospective nature, and a small sample size, necessitating univariate analysis (159).

Han et al. (160) performed an HR-MRI study in 51 adult MMD patients, analyzing angiographic and HR-MRI findings as well as atherogenic risk factors. The research group suggested that HR-MRI may assist in a precise diagnosis of ICAD in MMD patients with risk factors for atherogenesis. Distinguishable symptoms observed in moyamoya patients in the presence or absence of atherosclerotic plaques may suggest a distinct pathophysiology, implying different treatment strategies (160).

Storey et al. performed a single-center study which included 204 MMD patients. Transdural collateral vessels were existent in nearly 50% of arteriograms done preoperatively on these MMD patients. Pial synangiosis was performed on these patients from 2005 to 2013. This group hypothesized that preoperative transdural collateral vessels may be utilized as radiographic biomarkers of MMD, that transdural collateral vessels may be associated with advanced moyamoya, that transdural collateral vessels may be related to stroke as a perioperative complication, and that transdural collateral vessels may be capable of inducing surgical collaterals after surgery, suggesting innovative methods to generate biomarkers that may be capable of predicting outcome and stratifying surgical candidates, and supporting the usefulness of preoperative diagnostic arteriography (161).

Song et al. performed a retrospective analysis in 90 pediatric MMD patients by MRI and digital subtraction angiography to assess the distribution of ischemic lesions and vascular abnormalities, including stenosis and occlusion, as well as diagnostic coherence between imaging modalities. Digital subtraction angiography and MRA were used to detect stenotic and/or occlusive changes of the bilateral ACA, MCA, and PCA. MRI demonstrated good diagnostic coherence regarding stenotic and/or occlusive changes in the bilateral PCA. However, it is important to note that in 6 to 11% of patients, an MRA evaluation may lead to misdiagnosis of moyamoya (162).

Zhao et al. (31) conducted a single-center study to compare the radiographic and clinical characteristics between adult and pediatric moyamoya patients, and also between moyamoya patients with and without PCA involvement. Records of 696 consecutive MMD patients from 2009 to 2015, including 574 angiograms, 434 of adult and 140 of pediatric MMD patients, were reviewed. Grading systems by Miyamoto and Suzuki were used to evaluate stenoses and/or occlusions of the anterior and posterior cerebral circulation. In both pediatric MMD and in adult MMD, occlusive and/or stenotic PCA lesions positively correlated with the ipsilateral ICA stage. In adult MMD patients, the degree of PCA involvement seemed to correlate with the frequentness of ipsilateral stroke. In comparison to adult MMD patients, pediatric MMD patients showed a tendency toward advanced PCA lesions. First, this study is limited by its retrospective nature. Second, this study may be limited by selection bias (31).

In their 2019 review article, Li et al. stated that the most acceptable and valuable imaging modality should be reliable and keep injury to the patient to a minimum. Doppler and quantitative MRI-based imaging modalities might be auspicious in establishing an accurate evaluation of moyamoya. Standardized protocols, including various imaging modalities, to evaluate the preoperative and postoperative status quo, may be advocated in future moyamoya research (163).

Hauser et al. performed a retrospective study in 20 consecutive patients with angiographically proven MMD and analyzed 160 vascular territories, indicating that CO_2_-triggered blood oxygen level-dependent (BOLD) MRI may be a promising, easily implementable method for the evaluation of territorial hemodynamics in MMD patients with results comparable to those attained by [^15^O]H_2_O positron emission tomography/computed tomography with acetazolamide challenge, suggesting that, after continued prospective evaluation, CO_2_-triggered BOLD MRI may become a routine examination method in pre- and postoperative evaluation of MMD patients (164).

In their 2019 review article, Lehman et al. discuss novel MRI imaging achievements that may further improve the analysis of moyamoya, e.g., enhanced methods of cerebral perfusion and angiography, high-resolution characterization of the vessel wall, as well as artificial intelligence. In the clinical setting, contemporary and emerging MRI imaging methods may assist in the evaluation of MMD, and help guide therapy planning and response to treatment (165).

As vascular imaging methods advance in their potential to serve as research tools, our understanding of the pathophysiology of arterial stenosis in moyamoya is very likely to grow. These imaging advances are particularly important given the lack of an animal model that reproduces the spontaneous arterial stenosis of the proximal cerebrovasculature that is seen in moyamoya.

## Conclusion

Considerable progress has been made in moyamoya research since it was first described, leading to greater epidemiologic and pathophysiological understanding, as well as advancements in clinical practice, encompassing imaging, diagnosis, and surgical treatment. However, the proximate mechanisms that underly stenosis and progression of stenosis remain elusive. As a result, there is no clear drug target to reverse the disease process or prevent progression, and there have been no disease-modifying medical therapies developed to accomplish these goals. Such a therapy is desperately needed, as current medical therapies do not prevent progression and current surgical therapies, while effective, are associated with significant risk of morbidity and mortality. Future research should continue to aggressively pursue novel hypotheses that attempt to unify the unique aspects of moyamoya anatomic localization, pathophysiology, disease associations, and genetic risk factors to explain why stenosis develops in moyamoya. Further, as the understanding of more common stenotic arteriopathies of the distal ICA, such as atherosclerosis and FMD, is advanced, newly discovered mechanisms should continue to be investigated in moyamoya given its overlapping pathological characteristics. The initiation of arterial stenosis in moyamoya represents the biggest mystery of this disease process, and until it is mechanistically explained, patient care in moyamoya will remain insufficient.

## Author Contributions

BF and KD contributed to developing the concept of the review, developing the figures, and writing and editing the manuscript. ML oversaw the project and contributed to developing the concept of the review. JW led oversight of the project and contributed to developing the concept of the review, developing the figures, and editing the manuscript. All authors contributed to the article and approved the submitted version.

## Conflict of Interest

The authors declare that the research was conducted in the absence of any commercial or financial relationships that could be construed as a potential conflict of interest.

## Publisher's Note

All claims expressed in this article are solely those of the authors and do not necessarily represent those of their affiliated organizations, or those of the publisher, the editors and the reviewers. Any product that may be evaluated in this article, or claim that may be made by its manufacturer, is not guaranteed or endorsed by the publisher.

## Abbreviations

ACA: anterior cerebral artery
BOLD: blood oxygen level-dependent
HR-MRI: high-resolution magnetic resonance imaging
ICAD: intracranial atherosclerotic disease
MCA: middle cerebral artery
MMD: moyamoya disease
MMS: moyamoya syndrome
MRA: magnetic resonance angiography
MRI: magnetic resonance imaging
PCA, posterior cerebral artery. For a complete list of abbreviations and gene symbols: see Supplemental Table 1.
