# Corrigendum: Pathophysiology of Vascular Stenosis and Remodeling in Moyamoya Disease

**DOI:** 10.3389/fneur.2021.812027

**Published:** 2021-11-26

**Authors:** Brandon M. Fox, Kirsten B. Dorschel, Michael T. Lawton, John E. Wanebo

**Affiliations:** ^1^Department of Neurosurgery, St. Joseph's Hospital and Medical Center, Barrow Neurological Institute, Phoenix, AZ, United States; ^2^Medical Faculty, Heidelberg University Medical School, Ruprecht-Karls-Universität Heidelberg, Heidelberg, Germany

**Keywords:** angiopathy, cerebrovascular, moyamoya, stenosis, stroke

In the published article, there was an error in the Author Contributions Statement. The complete list of Brandon M. Fox and Kirsten B. Dorschel's contributions were not included. The corrected Author Contributions Statement appears below.

## Author Contributions

BF and KD contributed to developing the concept of the review, developing the figures, and writing and editing the manuscript. ML oversaw the project and contributed to developing the concept of the review. JW led oversight of the project and contributed to developing the concept of the review, developing the figures, and editing the manuscript. All authors contributed to the article and approved the submitted version.

The authors apologize for this error and state that this does not change the scientific conclusions of the article in any way. The original article has been updated.

## Publisher's Note

All claims expressed in this article are solely those of the authors and do not necessarily represent those of their affiliated organizations, or those of the publisher, the editors and the reviewers. Any product that may be evaluated in this article, or claim that may be made by its manufacturer, is not guaranteed or endorsed by the publisher.

