# Supplementary material for: Pathophysiology of Vascular Stenosis and Remodeling in Moyamoya Disease
Source: Front Neurol. 2021 Sep 3;12:661578. doi: 10.3389/fneur.2021.661578 (PMC8446194; doi:10.3389/fneur.2021.661578)
Supplement: Supplementary file 1 [file Table_1.DOCX]

**Supplemental Table 1.** Explanations of abbreviations and *gene symbols*

| **Abbreviation** | **Definition/Gene Name** | |
| --- | --- | --- |
| ACA | Anterior cerebral artery | |
| *ACTA2* | Actin alpha 2, smooth muscle | |
| *AKT1* | AKT serine/threonine kinase 1 | |
| *AKT2* | AKT serine/threonine kinase 2 | |
| *ATR* | ATR serine/threonine kinase | |
| BOLD | Blood oxygen level-dependent | |
| BP | Blood pressure | |
| *BRAF* | B-Raf proto-oncogene, serine/threonine kinase | |
| BRCC3 | Lys-63-specific deubiquitinase BRCC36 | |
| *BRCC3* | BRCA1/BRCA2-containing complex subunit 3 | |
| *CENPJ* | Centromere protein J | |
| *CEP152* | Centrosomal protein 152kDa | |
| *CEP63* | Centrosomal protein 63kDa | |
| ERK | Extracellular signal-regulated kinase | |
| GUCY1A3 | Guanylate cyclase 1 soluble subunit alpha 3 | |
| *GUCY1A3* | Guanylate cyclase 1 soluble subunit alpha 3 | |
| *HRAS* | Harvey rat sarcoma viral oncogene homolog | |
| HR-MRI | High-resolution magnetic resonance imaging | |
| ICAD | Intracranial atherosclerotic disease | |
| ICASO | Intracranial major artery stenosis/occlusion | |
| *JAG1* | Jagged canonical Notch ligand 1 | |
| *KRAS* | KRAS proto-oncogene, GTPase | |
| *MAP2K1* | Mitogen-activated protein kinase kinase 1 | |
| MCA | Middle cerebral artery | |
| MMD | Moyamoya disease |  |
| MMS | Moyamoya syndrome |  |
| MRA | Magnetic resonance angiography |  |
| MRI | Magnetic resonance imaging |  |
| MTCP1 | Protein p13 MTCP-1 |  |
| *MTCP1* | Mature T-cell proliferation 1 |  |
| NF-1 | Neurofibromatosis type I |  |
| *NF1* | Neurofibromin 1 |  |
| *NIN* | Ninein |  |
| NO-sGC-cGMP | Nitric oxide-soluble guanylyl cyclase-cyclic guanosine monophosphate |  |
| NO | Nitric oxide |  |
| Notch | Neurogenic locus notch homolog protein |  |
| *NOTCH2* | Notch receptor 2 |  |
| *NRAS* | NRAS proto-oncogene, GTPase |  |
| PCA | Posterior cerebral artery |  |
| *PCNT* | Pericentrin |  |
| PHACES syndrome | Posterior fossa malformations, hemangiomas, arterial anomalies, cardiac defects, eye abnormalities, sternal cleft, and supraumbilical raphe syndrome |  |
| PIRAMD | Prior infarcts, reactivity, and angiography in moyamoya disease |  |
| *PTPN11* | Protein tyrosine phosphatase non-receptor type 11 |  |
| *RAF1* | Proto-oncogene C-RAF |  |
| Ras-Raf-MEK-ERK | Rat sarcoma/rat fibrosarcoma/mitogen-activated protein kinase kinase/extracellular signal-regulated kinase |  |
| *RBBP8* | RB binding protein 8, endonuclease |  |
| *SOS1* | SOS Ras/Rac guanine nucleotide exchange factor 1 |  |
| TIA | Transient ischemic attack |  |
